# Investigation of the Impact of Damaged Smartphone Sensors’ Readings on the Quality of Behavioral Biometric Models

**DOI:** 10.3390/s22249580

**Published:** 2022-12-07

**Authors:** Paweł Rybka, Tomasz Bąk, Paweł Sobel, Damian Grzechca

**Affiliations:** 1 Digital Fingerprints, ul. Żeliwna 38, 40-599 Katowice, Poland; 2 Faculty of Automatic Control, Electronics and Computer Science, Silesian University of Technology, ul. Akademicka 16, 44-100 Gliwice, Poland

**Keywords:** behavioral biometrics, machine learning, MEMS, Multi-Factor Authentication

## Abstract

Cybersecurity companies from around the world use state-of-the-art technology to provide the best protection against malicious software. Recent times have seen behavioral biometry becoming one of the most popular and widely used components in MFA (Multi-Factor Authentication). The effectiveness and lack of impact on UX (User Experience) is making its popularity rapidly increase among branches in the area of confidential data handling, such as banking, insurance companies, the government, or the military. Although behavioral biometric methods show a high degree of protection against fraudsters, they are susceptible to the quality of input data. The selected behavioral biometrics are strongly dependent on mobile phone IMU sensors. This paper investigates the harmful effects of gaps in data on the behavioral biometry model’s accuracy in order to propose suitable countermeasures for this issue.

## 1. Introduction

According to the FBI’s (Federal Bureau of Investigation) 2021 report on Internet crime [1], the number of phishing attacks reported to the IC3 (Internet Crime Complaint Center) doubled in 2021 (241,342 incidents) compared to 2020 (114,702 incidents) and was almost ten-times higher than in 2019 (26,379 incidents). This data clearly shows how popular phishing attacks [2,3] are and how the demand for phishing countermeasures is growing in the government, banking, and military sectors. Although a multitude of companies and governments organize staff training on cybersecurity, accounts are still being hacked as fraudster attacks become smarter and better targeted [4,5]. Fortunately, even if the user’s login and password have been voluntarily provided to the fraudster, there are still a number of ways to protect accounts against being hijacked; one of them is behavioral biometrics.

Smartphone sensors (for example, accelerometers, magnetometers, gyroscopes) find a lot of applications when it comes to mobile apps—from entertainment (mobile games) to monitoring user’s behavior (pedometers, sleep monitoring, and others). Recently, the use of smartphone sensors has found applications in more advanced systems such as health monitoring [6] or cybersecurity. As the consumption of multimedia and mobile resources access rises day-to-day [7], the topic of behavioral biometry and its impact on cybersecurity has recently been present in many research papers covering both desktop [8] and mobile [9] device usage.

The latest studies [10,11] have shown that the use of behavioral biometrics has become an increasingly popular part of MFA (Multi-Factor Authentication) [12]. Over the last couple of years, institutions for confidential data handling have been more prone to reach for users’ behavioral patterns (e.g., keyboard strokes, mouse movements, or mobile device handling) when implementing identity theft countermeasures, as this sort of data does not require any additional user involvement harmful for the UX (User Experience) [13]. Moreover, behavioral biometric models show high resistance to fraudsters as their behavior is vastly different from what users tend to do; as such, the combination of suspicious activity on user’s account (e.g., a transfer for a high amount) with unusual keyboard or smartphone readings may indicate an attack [14,15].

Although the individual user behavioral biometrics model is a powerful weapon against account hijacking, it is still vulnerable to low data quality delivered from used devices. Swapping mobile phones or damaging certain sensors may lead to reduced quality of fraud detection. For the sake of maintaining the high quality of behavioral biometry authentication services, it is a must to implement precautionary rules. This paper compares how exemplary behavioral models based on aggregated accelerometer and gyroscope readings deal with incomplete anonymized user data. The first stage for applying quality drop countermeasures would be labeling certain users’ behavioral data readings as “low quality” to prevent data damage that reduces the model’s classification accuracy below established thresholds. The next stages would be more complex—for example, providing user models resistant to sensor damage or applying additional safe mode pipelines.

The most recent papers published on the matter of behavioral biometrics show experimental attitudes towards data and introduce results which do not cover real-life scenario difficulties. The following analysis presents a real-life industrial-based case study on behavioral authentication for one of the leading national banks. This paper covers all the data processing pipeline issues and problems regarding the quality and quantity of certain users’ data as well as the struggle encountered with the use of multiple devices and OS versions.

At the beginning of the manuscript, the dataset is introduced; then, the mathematical background of the behavioral model (input vectors, structure, hyperparameters etc.) is presented. In the end, the models’ input vectors are artificially disturbed with commonly occurring data damage and their quality is examined to verify whether the presented solution is susceptible to certain types of data absence.

The related papers the authors highly recommend getting familiarized with are [16], where researchers present a proposal of a continuous authentication system for smartphone user classification based on interactions with the device, and [17], which provides BehavePassDB—a public database for mobile behavioral biometrics solution benchmarking. This database can be used as a sandbox for testing new features and classification algorithms before feeding further data. The use of behavioral biometry in mobile devices provides reliable security for zero price when UX is considered, and numerous researchers emphasize the importance of maintaining UX of the highest quality [18]. It is the clients themselves (banks and other institutions covered by behavioral biometry cybersecurity solutions) that insist on keeping the system user-friendly. Additionally, as the global COVID-19 pandemic and its repercussions caused severe changes in the use of digital resources, behavioral biometry has proven to be a high-quality cyberattack countermeasure in fields where other security systems have failed [19].

## 2. Materials and Methods

**Figure 1:**
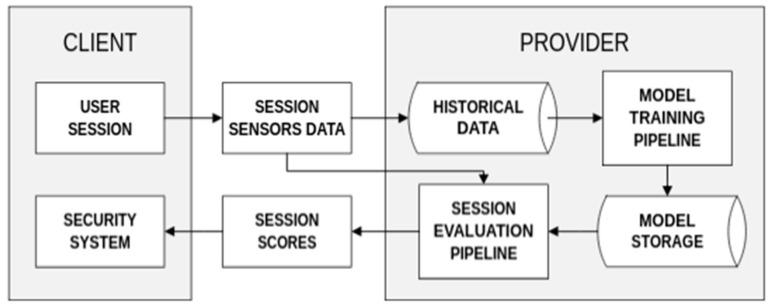
Data exchange pipeline for the behavioral biometry security system.

The data used for this research was acquired from the accelerometer and gyroscope sensors of smartphone devices running banking applications on the Android operating system [20], coming from users of one of the leading national banks. Sensor readings were collected from the beginning until the end of use of the banking application. In this case, the data was sent to the upstream node and evaluated in real time. Whenever fraudulent behavior is detected at any stage of the session, an alert signal is sent to the mobile application provider (usually the bank’s department of security). The alerting system does not take any additional meta-data apart from an historical behavioral profile. No information on age, gender, banking history, device type, OS, or other data is stored or analyzed. The following block diagram presents how the information exchange between the client and the provided security system is organized (Figure 1):

### 2.1. Dataset Structure

Users u perform a certain number $SN_{u}$ of connections called sessions $S^{u}$ (1) with the server via the banking application. Each user session $s_{i}^{u}$ that lasts for $TS_{s_{i}^{u}}$ seconds, consists of feature vectors whose count is a number denoted by $FC_{s_{i}^{u}}$ (2). Every single user feature vector D in the entire population consists of a fixed number (N = 6) of features d (3). The feature vector used for the analysis was formed in the following manner: accelerometer *x* axis; accelerometer *y* axis; accelerometer *z* axis; gyroscope *x* axis; gyroscope *y* axis; gyroscope *z* axis.

(1) $$S^{u}=\left[s_{1}^{u},\;s_{2}^{u},\;\ldots ,\;s_{k}^{u},\;\ldots ,\;s_{SN_{u}}^{u}\right]$$

(2) $$s_{i}^{u}=\left[D_{1},\;D_{2},\;\ldots ,\;D_{FC_{s_{i}^{u}}}\right]$$

(3) $$D_{i}=\left[d_{1},\;d_{2},\;\ldots ,\;d_{N}\right]$$

For the sake of applying user classification, the sensor readings from a single session $s_{i}^{u}$ are aggregated column-wise into windows W of intervals WI = 20 s (4). Each element of window vector *W* denotes a single window where all feature vectors from a certain interval are stored. The sampling frequency is not uniform and varies based on the user’s device and data pipeline processing issues (e.g., packet losses). The aggregates are responsible for converting the data into smaller chunks and for immunizing it from being sampling frequency-susceptible.

(4) $$W=\left[W_{1},\;W_{2},\;\ldots ,\;W_{N_{W}}\right],\;where\;N_{W}=\lfloor \frac{{TS}_{s_{i}^{u}}}{WI}\rfloor$$

$t_{0}$ indicates the session starting time and $t_{D_{x}}$ indicates the time which passed since $t_{0}$ until the creation of a feature vector $D_{x}$ (5). The number of all feature vectors in a single window is denoted by $N_{M}$ (6).

(5) $$W_{j}=s_{i}^{u},where\lfloor \frac{t_{D_{x}}}{WI}\rfloor =j$$

(6) $$W_{j}=\left[w_{1},{\;w}_{2},\;.\ldots ,{\;w}_{N_{M}}\right]$$

The following Formulas (7)–(9) show how the aggregated features vector F is created:

(7) $$W_{j}^{k}=\left[w_{1}\left(k\right),\;w_{2}\left(k\right),\;\ldots ,\;w_{N_{M}}\left(k\right)\right]\;$$

(8) $$F=\left[f_{1},\;f_{2},\;\ldots ,\;f_{4N}\right]$$

(9) $$f_{k}=aggregate_{k\;mod\;4}\left(W_{j}^{k}\right)$$

The total length of the aggregated feature vector equals the length of the original feature vector times the number of all the aggregating methods (8)—standard deviation, arithmetic mean, amplitude, and median—which is denoted by 4N. The formula on which the aggregates are based is shown below (10):

(10) $$\begin{array}{c} aggregate_{type}\left(X\right)\\ =\{\begin{array}{cc} \frac{1}{P}\overset{P}{\underset{j=1}{\sum }}{\left(x_{j}-\mu \right)}^{2},\;where\;\mu =\frac{1}{P}\overset{P}{\underset{j=1}{\sum }}\left(x_{i}\right), & for\;type=0\\ \frac{1}{P}\overset{P}{\underset{j=1}{\sum }}\left(x_{j}\right), & for\;type=1\\ \max\left(X\right)-\min\left(X\right) & for\;type=2\\ sx_{\frac{P}{2}}\;if\;P\;mod\;2=0,\frac{sx_{\frac{\left\lfloor P\right\rfloor }{2}}+sx_{\frac{\left\lfloor P\right\rfloor }{2}+1}}{2}\;if\;P\;mod\;2=1\;where\;SX=sort\left(X\right) & for\;type=3 \end{array} \end{array}$$

### 2.2. Data Preparation

**Figure 2:**
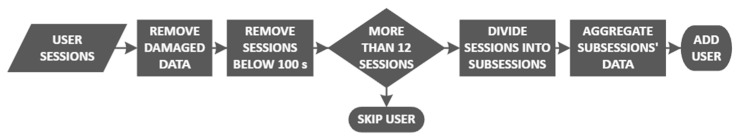
The user choice rules pipeline.

**Figure 3:**
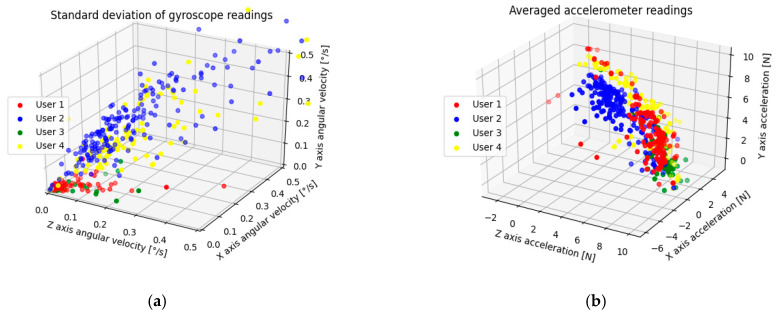
Exemplary user data on average accelerometer readings (**a**) and standard deviation of gyroscope readings (**b**) from 20-second intervals.

In order to ensure reliable training and testing datasets, only those users with more than a certain number of unique sessions ($SN_{u}>12$), and ones for which a model could be built (undamaged data) were considered. Each viable session had to last for a fixed amount of time $TS_{min}$ or longer ($TS_{s_{i}^{u}}$ > $TS_{min}$ = 100 s) to assure the occurrence of at least $WC_{min}$ = 5 windows lasting for WI. Each window generated a sub-score; further sub-score processing resulted in score generation. The user choice rule described above is presented in a flow chart below (Figure 2):

From a total of 264 users, only 127 fulfilled the requirements (118 users did not meet data quantity needs and 19 users failed training, resulting in generation of a low-quality model). The exemplary aggregate ***W*** of the sensor readings of 4 random users are presented in Figure 3.

Each session consisted of at least 5 sub-scores, varying from 0 to 1 (indicating the similarity measure coming from the classifier’s output)—to indicate that the behavior was user-like. In order to correctly evaluate the session, the final score—consisting of M sub-scores—was calculated as their average (11). Whenever the final score exceeded the user-defined threshold (evaluated based on certain business requirements of the bank), the session was considered fraudulent (12).

(11) $$score=\frac{\sum_{i=1}^{M}subscore_{i}}{M},\;where\;M\geq 5$$

(12) $$assignment=\{\begin{array}{cc} 0\;\left(user\right), & is\;score<thr\\ 1\;\left(fraudster\right), & if\;score\geq thr \end{array}$$

**Figure 4:**
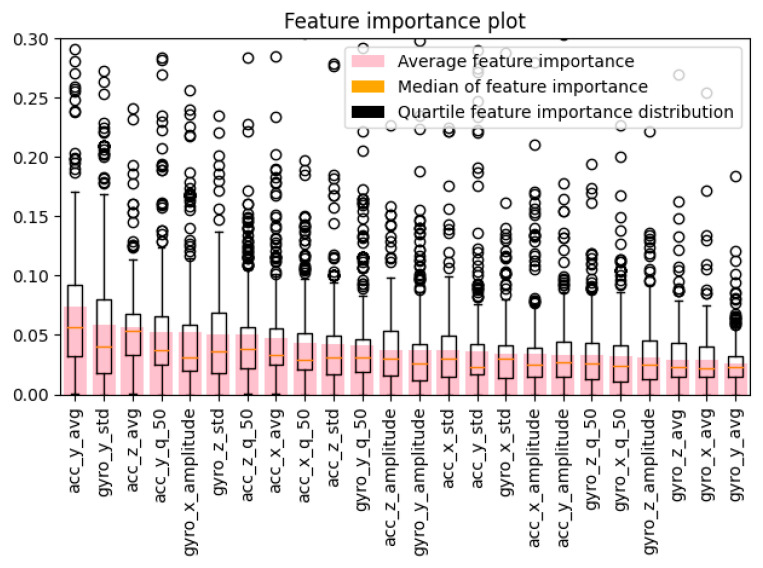
Feature importance of aggregated data (averages, standard deviations, medians, and amplitudes) of the *x*, *y*, and *z* axes of the accelerometer and gyroscope readings.

To provide in-depth data insight, the feature importance [21,22,23] for 24 aggregates was calculated. The feature significance was estimated using the XGBoost (eXtreme Gradient Boosting) classification algorithm, with its hyperparameters heuristically optimized. [24]. The boxplot below (Figure 4) shows the feature importance distribution, sorted by descending average importance.

**Figure 5:**
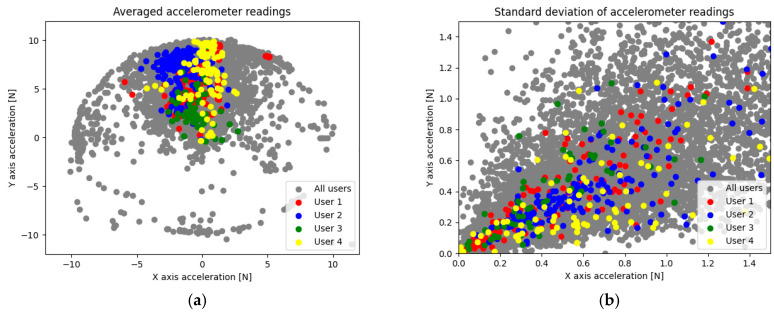
Average (**a**) and standard deviation (**b**) of accelerometers’ *x* and *y* axes (all users).

**Figure 6:**
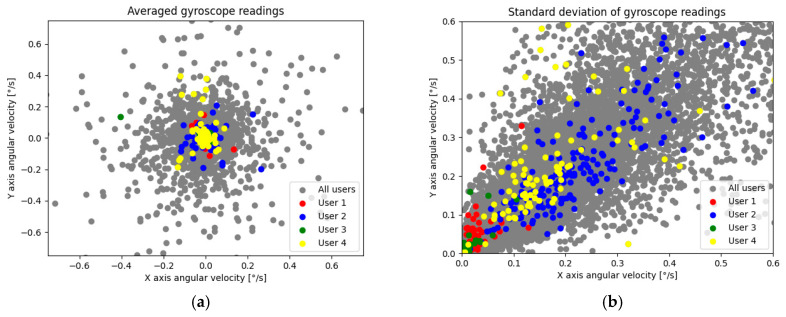
Average (**a**) and standard deviation (**b**) of gyroscopes’ *x* and *y* axes (all users).

By analyzing the data presented in Figure 2, we can notice a relationship—the accelerometer data worked best with averages while the gyroscope provided the best diagnostic value (higher average and median feature importance) with standard deviations. This is caused by the nature of the data provided by those sensors; a certain user distinguishability depending on the sensor type and aggregation method can be seen in Figure 5 (accelerometer) and Figure 6 (gyroscope). In order to provide a clearer visualization, 4 randomly chosen users were highlighted.

### 2.3. XGBoost Training

**Figure 7:**
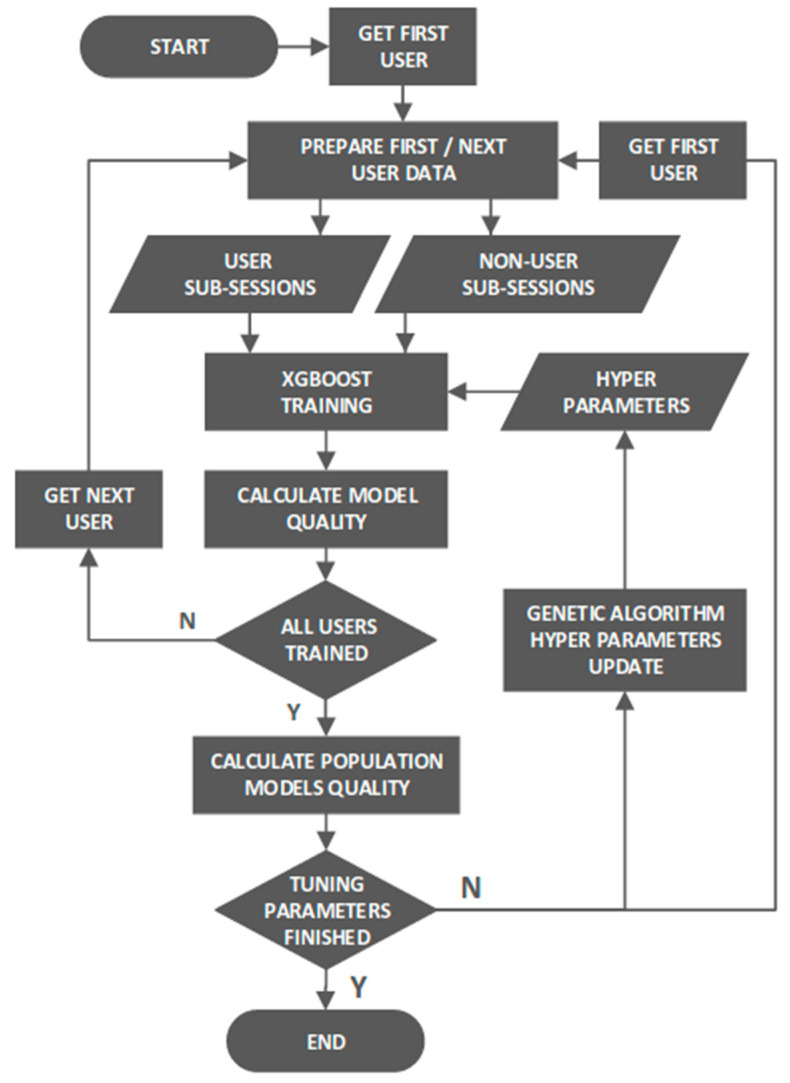
Flow chart representing the XGBoost classifier hyperparameter optimization process.

To train a model, the user’s data (sub-sessions) were labeled as an authorized session and the data considering the remaining 126 users were labeled as a fraudulent one. Subsequently, the data set was passed to the XGBoost classifier—the boosting estimator based on decision trees [25,26,27,28,29,30], in which the trees are expanded to a forest where each estimator is built on the residual value of the previous classification. To reach the best possible model, the XGBoost’s hyperparameters were tuned for the whole population [31]. The method used for reaching the best possible model quality is presented in a flow chart in Figure 7. The method trains users stored in a queue with certain hyperparameters set. When all the users are trained, their mean model quality is calculated and is treated as fitness. This procedure was reproduced a fixed number of times with a different set of hyperparameters (which came from the evolutionary algorithm). The best hyperparameter set did not change and was treated as the target set.

**Figure 8:**
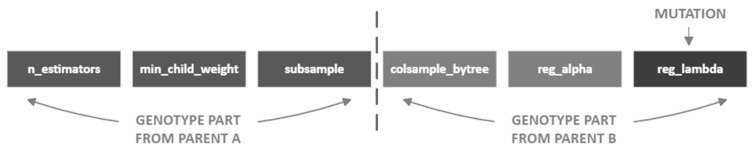
Exemplary hyperparameter child genotype from the evolutionary algorithm.

The classifier hyperparameters were optimized using an evolutionary algorithm whose outcome is presented in Table 1 below (non-listed parameters were set to default). The evolutionary algorithm started with a population of 10 randomly chosen values of 6 hyperparameters taken randomly from the uniform distribution, with upper and lower boundaries denoted as “min” and “max” in Table 1) and performed 10 steps of vector crossing (selecting 2 equal subparts of two different hyperparameters’ dictionaries and combining them) and a one-value mutation (randomly reselecting a particular parameter’s value from the specified domain), leaving only the top set of hyperparameter values at each step. Its fitness function was the same as the classifier’s objective function, denoted by Formula (13). The vector that provided the best model qualities among the entire population was kept for further consideration. The exemplary genotype division and mutation are presented in Figure 8.

The model validation data set consists of 30% of the total data. To reproduce real-life model usage, as well as to prevent data leakage, the evaluation was run only on the most recent readings. The objective function used for the model training is represented by Formula (13), where TP stands for “true positive” prediction, TN for “true negative”, FP for “false positive”, and FN for “false negative”:

(13) $$fitness=e^{\frac{\log\left(\frac{TP}{TP+FN}\right)*\log\left(\frac{TN}{TN+FP}\right)}{2}}$$

**Figure 9:**
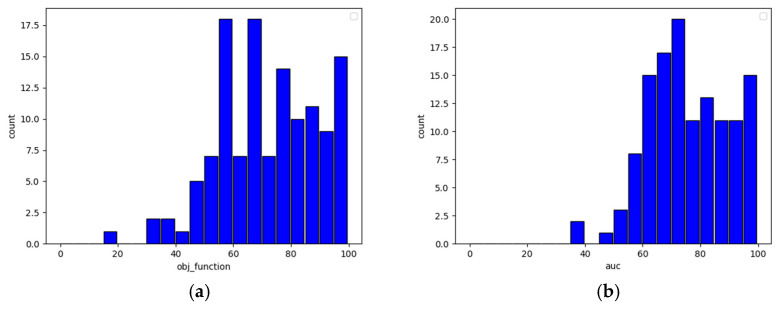
Model quality distribution for all users—objective function (**a**) and ROC-AUC (**b**).

To illustrate what an undamaged sensor’s model quality looks like, let us examine Figure 9, showing the model quality distribution in terms of the objective function and the receiver operating characteristic’s area under the curve (ROC-AUC) for the entire population. The higher the value of both the objective function and the receiver operating characteristic, the better the model quality is. Any damage done to models (for example, by applying data with missing values for training) will result in the decay of both metrics.

## 3. Results

The experiment investigating the harmful effects of sensor damage on behavioral biometrics model quality was run by replacing certain axis data with zero values. The idea behind conducting such an analysis derives from the necessity of knowing whether the model output is still valid, meaning the authentication provided by the behavioral biometry can be trusted. For minor damage—e.g., one gyroscope axis—the model could still provide valuable information, while deleting the data coming from all the axes may cause the model to become utterly useless. To find those boundaries, the two most common data collection failures were considered: damaging the data from one axis of the sensor and damaging the data from all axes of the sensor. Table 2 provides information on how average models’ quality decays due to the zeroing of certain vector values.

**Figure 10:**
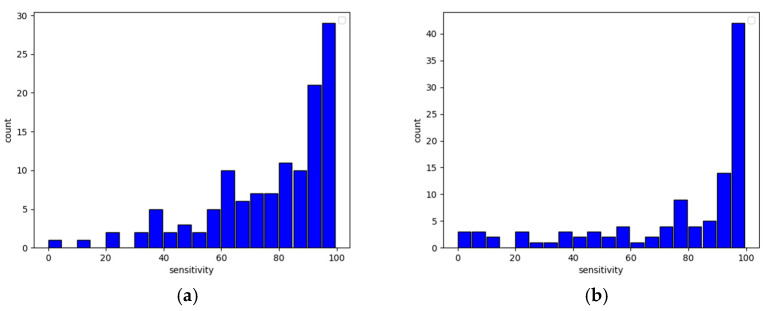
User sensitivity histogram with undamaged data (**a**) and data without accelerometer axis *z* (**b**).

**Figure 11:**
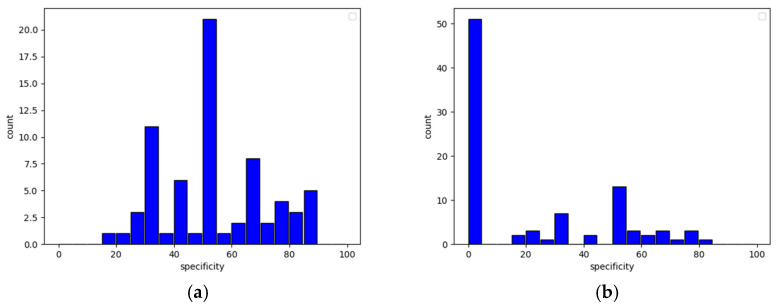
User specificity histogram with undamaged data (**a**) and data without accelerometer axis *z* (**b**).

The presented table explicitly proves that the lack of even one axis may severely damage the quality of predictions. What is also worth noticing is that the true negative rate heavily dropped due to data changes while the true positive rate remained almost the same. Such behavior causes models to produce an increased amount of false positive output, and this leads to overwhelming of the system with false fraudster alerts (correct user detection is in most cases the same though). The histograms presented in Figure 10 (sensitivity) and Figure 11 (specificity) clearly show this relationship—the higher the specificity, the less likely the model is to classify a fraudster as a user, and the higher the sensitivity, the more reliable user detection becomes.

## 4. Discussion

The presented paper investigates commonly occurring issues with mobile sensor data used for behavioral biometry. To properly approach faulty data handling (while detecting, e.g., zero values on a certain axis), it is necessary to know the model quality drop for particular-axis damage. The results of the analysis showed that when certain sensor axis data is missing, then vectors used for user authentication may cause a major drop in model accuracy. What is worth noticing and what derives from both the feature importance plot and from the damage influence table is the fact that the most harmful factor for user identification is the loss of accelerometer readings (especially the *z* axis—34 p.p. compared to the original objective function). Such damage causes the same ROC-AUC drop as losing all the gyroscope’s readings (ROC-AUC lowered by 0.15). This is caused by the fact that the accelerometer’s *z* axis provides user-distinctive data—it is highly responsible for indicating at what position the smartphone or tablet is held by the user and how his/her grip changes over time. On the other hand, when it comes to the gyroscope’s *z* axis, this parameter holds the lowest amount of user-distinctive data (objective function only dropping by 9 p.p.). This may be caused by the fact that there exists no substantial angular movement in this axis, or because all the movements are repeatable over the entire population.

## 5. Conclusions

**Figure 12:**
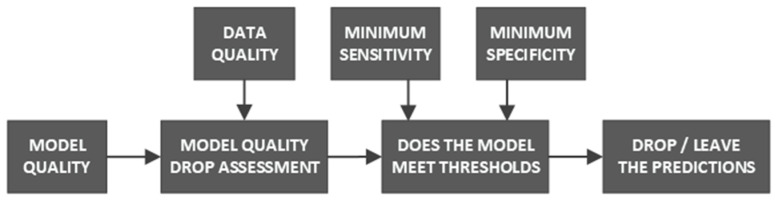
Prediction quality assessment pipeline proposal.

The true negative ratio is the most affected metric, and this means that the model’s ability to correctly distinguish a user from the rest of the population will not work well—the model will classify user sessions as fraudulent ones. Such behavior will heavily deteriorate security systems by setting off false alerts. To prevent this, we can change the classification threshold level by increasing specificity at the cost of sensitivity; yet, this approach will not improve the total model accuracy. These minor specificity drops should be compensated for by increasing the classification threshold by a predefined factor (which will result in increases in objective function), while higher drops should raise a flag indicating that the evaluation score is invalid. A different approach to dealing with lower-quality models rests with decreasing their weights in an authentication system. Usually, authorization via behavioral biometry uses several models that measure several types of activity—if we can assess a certain model’s quality for certain data, we can lower the contribution provided by this model in generating the final score. Yet another way of evading quality drop is using different classifiers—those less susceptible to data damage or those using data preprocessing methods that immunize models against data damage. We can as well think of modifying the classification pipeline—whenever nothing but zero values are present on a specific axis, we can decide whether to evaluate the session or to skip the evaluation (for example, the authentication process is run only if sensitivity did not drop below 70% and specificity did not drop below 60% after introducing certain-axis damage). The additional session evaluation block would take model statistics, as well as a data structure, and assess the prediction reliability (Figure 12).

What may be worth noticing is that further examination of the model quality drop can be used to estimate numerous device/user-related issues, e.g., to identify device damage. If the user did not show any symptoms of classification problems, and after some time generates numerous false alerts, we may conclude that the sensors do not work correctly anymore or that there are different issues (e.g., user illness or malware attack). These assumptions, however, require further studies.

Future work will mostly be focused on building high-quality damaged-sensor handling pipelines. In case of damaged data occurrence, we must be able to quickly assess the quality of incoming information and to find an efficient way of detecting session hijacking, even if the most valuable information is lost due to data collection errors.

Additional study directions to take should be focused on immunizing the system to erroneous data as well as improving the system’s overall quality by introducing novel noise-resistant classifiers and data processors. What should also be kept in mind is the fact that different devices (keyboard or mouse) and different features may not respond similarly to what the analysis has shown. Quality drops caused by missing data be separately examined and countermeasures introduced to them may differ from the ones implemented for mobile devices.

## Figures and Tables

**Table 1:** XGBoost classifier hyperparameters optimized with the use of a genetic algorithm.

Hyperparameter	Value	Min	Max	Description
n_estimators	220	1	350	Number of weak classifiers (gradient-boosted trees)
min_child_weight	7	2	7	Minimum sum of instance weight (hessian) needed in a child
subsample	0.658	0.1	0.99	Subsample ratio of the training instance
colsample_bytree	0.791	0.5	1.0	Subsample ratio of columns when constructing each tree
reg_alpha	0.415	0.0	2.0	L1 regularization term on weights
reg_lambda	0.566	0.0	2.0	L2 regularization term on weights

**Table 2:** The influence of sensor data damage on average model quality.

Zeroed Feature	Sensitivity (True Positive Rate)	Specificity (True Negative Rate)	Objective Function	ROC-AUC
None (undamaged data)	0.78	0.74	0.72	0.76
Accelerometer (*x* axis)	0.71	0.65	0.56	0.68
Accelerometer (*y* axis)	0.70	0.61	0.50	0.66
Accelerometer (*z* axis)	0.80	0.43	0.38	0.61
Accelerometer (*x*, *y*, *z* axes)	0.71	0.33	0.14	0.52
Gyroscope (*x* axis)	0.77	0.61	0.58	0.69
Gyroscope (*y* axis)	0.78	0.60	0.58	0.69
Gyroscope (*z* axis)	0.77	0.68	0.63	0.72
Gyroscope (*x*, *y*, *z* axes)	0.77	0.45	0.38	0.61

## Data Availability

Not applicable.

## References

[1] (2020). Internet Crime Report. Internet Crime Complaint Center (IC3). https://www.ic3.gov/Media/PDF/AnnualReport/2020_IC3Report.pdf.

[2] Desolda G., Ferro L.S., Marrella A., Catarci T., Costabile M.F. (2022). Human Factors in Phishing Attacks: A Systematic Literature Review. ACM Comput. Surv..

[3] Alkhalil Z., Hewage C., Nawaf L., Khan I. (2021). Phishing Attacks: A Recent Comprehensive Study and a New Anatomy. Front. Comput. Sci..

[4] Shahbaznezhad H., Kolini F., Rashidirad M. (2021). Employees’ Behavior in Phishing Attacks: What Individual, Organizational, and Technological Factors Matter?. J. Comput. Inf. Syst..

[5] Aneke J., Ardito C., Desolda G. (2021). Help the User Recognize a Phishing Scam: Design of Explanation Messages in Warning Interfaces for Phishing Attacks. Proceedings of the International Conference on Human-Computer Interaction.

[6] Majumder S., Deen M.J. (2019). Smartphone Sensors for Health Monitoring and Diagnosis. Sensors.

[7] Falkowski-Gilski P. (2020). On the Consumption of Multimedia Content Using Mobile Devices: A Year to Year User Case Study. Arch. Acoust..

[8] Teh P.S., Teoh A.B.J., Yue S. (2013). A Survey of Keystroke Dynamics Biometrics. Sci. World J..

[9] Stylios I., Kokolakis S., Thanou O., Chatzis S. (2021). Behavioral biometrics & continuous user authentication on mobile devices: A survey. Inf. Fusion.

[10] Sahdev S.L., Singh S., Kaur N., Siddiqui L. Behavioral Biometrics for Adaptive Authentication in Digital Banking—Guard Against Flawless Privacy. Proceedings of the 2021 International Conference on Innovative Practices in Technology and Management (ICIPTM).

[11] Almalki S., Assery N., Roy K. (2021). An Empirical Evaluation of Online Continuous Authentication and Anomaly Detection Using Mouse Clickstream Data Analysis. Appl. Sci..

[12] Ometov A., Bezzateev S., Mäkitalo N., Andreev S., Mikkonen T., Koucheryavy Y. (2018). Multi-Factor Authentication: A Survey. Cryptography.

[13] Chalhoub G., Flechais I., Nthala N., Abu-Salma R., Tom E. Factoring User Experience into the Security and Privacy Design of Smart Home Devices: A Case Study. Proceedings of the Extended Abstracts of the 2020 CHI Conference on Human Factors in Computing Systems.

[14] Matsuoka K., Irvan M., Kobayashi R., Yamaguchi R.S. A Score Fusion Method by Neural Network in Multi-Factor Authentication. Proceedings of the Tenth ACM Conference on Data and Application Security and Privacy.

[15] Miyazawa A., Thao T.P., Yamaguchi R.S. Multi-factor Behavioral Authentication Using Correlations Enhanced by Neural Network-based Score Fusion. Proceedings of the 2022 IEEE 19th Annual Consumer Communications & Networking Conference (CCNC).

[16] Rocha R., Carneiro D., Costa R., Analide C., Novais P., Lloret J., Chamoso P., Carneiro D., Navarro E., Omatu S. (2020). Continuous Authentication in Mobile Devices Using Behavioral Biometrics. Proceedings of the Ambient Intelligence—Software and Applications—10th International Symposium on Ambient Intelligence.

[17] Stragapede G., Vera-Rodriguez R., Tolosana R., Morales A. (2023). BehavePassDB: Public Database for Mobile Behavioral Biometrics and Benchmark Evaluation. Pattern Recognit..

[18] Falkowski-Gilski P., Stefański J. (2015). Quality Expectations of Mobile Subscribers. J. Telecommun. Inf. Technol..

[19] Chyzhevska M., Romanovska N., Ramskyi A., Venger V., Obushnyi M. Behavioral Biometry as a Cyber Security Tool. Proceedings of the Cybersecurity Providing in Information and Telecommunication Systems II.

[20] Falkowski-Gilski P., Stefański J. (2015). Android OS: A Review. Tem J..

[21] Cenggoro T.W., Mahesworo B., Budiarto A., Baurley J., Suparyanto T., Pardamean B. (2019). Features Importance in Classification Models for Colorectal Cancer Cases Phenotype in Indonesia. Procedia Comput. Sci..

[22] Chen C., Shi H., Jiang Z., Salhi A., Chen R., Cui X., Yu B. (2021). DNN-DTIs: Improved drug-target interactions prediction using XGBoost feature selection and deep neural network. Comput. Biol. Med..

[23] Muslim M.A., Dasril Y. (2021). Company bankruptcy prediction framework based on the most influential features using XGBoost and stacking ensemble learning. Int. J. Electr. Comput. Eng. (IJECE).

[24] Putatunda S., Rama K. A Comparative Analysis of Hyperopt as Against Other Approaches for Hyper-Parameter Optimization of XGBoost. Proceedings of the 2018 International Conference on Signal Processing and Machine Learning.

[25] Ogunleye A.A., Wang Q.-G. (2020). XGBoost Model for Chronic Kidney Disease Diagnosis. IEEE/ACM Trans. Comput. Biol. Bioinform..

[26] Dhaliwal S.S., Nahid A.-A., Abbas R. (2018). Effective Intrusion Detection System Using XGBoost. Information.

[27] Jing X., Zou Q., Yan J., Dong Y., Li B. (2022). Remote Sensing Monitoring of Winter Wheat Stripe Rust Based on mRMR-XGBoost Algorithm. Remote Sens..

[28] Sanders W., Li D., Li W., Fang Z.N. (2022). Data-Driven Flood Alert System (FAS) Using Extreme Gradient Boosting (XGBoost) to Forecast Flood Stages. Water.

[29] Liu Y., Wang H., Fei Y., Liu Y., Shen L., Zhuang Z., Zhang X. (2021). Research on the Prediction of Green Plum Acidity Based on Improved XGBoost. Sensors.

[30] Shahbazi Z., Byun Y.-C. (2022). Knowledge Discovery on Cryptocurrency Exchange Rate Prediction Using Machine Learning Pipelines. Sensors.

[31] Chen J., Zhao F., Sun Y., Yin Y. (2020). Improved XGBoost model based on genetic algorithm. Int. J. Comput. Appl. Technol..

